# Innervation zone distribution of the biceps brachii muscle examined using voluntary and electrically-evoked high-density surface EMG

**DOI:** 10.1186/s12984-019-0544-6

**Published:** 2019-06-11

**Authors:** Chengjun Huang, Cliff S. Klein, Zhaojian Meng, Yingchun Zhang, Sheng Li, Ping Zhou

**Affiliations:** 1 Guangdong Work Injury Rehabilitation Center, Guangzhou, Guangdong China; 2 Department of Physical Medicine and Rehabilitation, University of Texas Health Science Center at Houston, Houston, TX USA; 3 TIRR Memorial Hermann Research Center, 1333B Moursund St, TIRR Research Building, Suite 326, Houston, TX 77030 USA; 4 Department of Biomedical Engineering, University of Houston, Houston, TX USA

**Keywords:** EMG, Innervation zone, High-density electrode matrices, Electrical stimulation, Voluntary contraction

## Abstract

**Background:**

High density surface electromyography (EMG) can be used to estimate muscle innervation zones (IZ). The objective of this study was to compare the differences in the distribution of the biceps brachii (BB) IZ derived from voluntary contractions (VC) and electrical stimulation (ES) of the musculocutaneous nerve.

**Methods:**

Surface EMG signals were recorded from the medial and lateral BB with two 64-channel high density electrode matrices in eight healthy men. The surface EMG was recorded at different percentages of the maximal voluntary contraction (MVC) force (20–100% MVC) and at different percentages of the current needed to elicit a maximal M-wave (20–100% I_max_). The IZs of the medial and lateral BB were identified from the EMG signals and expressed as a row number within a given medial-lateral column.

**Results:**

ES current intensity had no significant effect on the group mean IZ location (*p* > 0.05). However, The IZ during VC was located more proximally with increasing force (*p* < 0.05), likely due to muscle shortening. The position of the IZ varied slightly (by up to ~ 8 mm) in a medial-lateral direction under both contraction types, but this spatial effect was not significant. The IZ during ES and weak VC (20, 40% MVC) was similar (*p* > 0.05), but was more proximal in the latter than the former during 60–100% MVC (*p* < 0.05).

**Conclusion:**

ES can be used to detect spatial differences in IZ location free of the confounding effects of muscle shortening and recruitment order of different sized motor units. The method may prove beneficial for locating the IZ in patients who lack voluntary control of their musculature.

## Introduction

Muscle spasticity often occurs in patients with neurological damage such as stroke and can have a negative impact on motor function [1]. It has been demonstrated that intramuscular injection of botulinum neurotoxins (BTX) is an effective and relatively safe treatment for spasticity [2, 3]. However, there are side effects including muscle weakness, blocking of autonomic nerves, and muscle atrophy, which may relate to the toxin dosage [4]. The effectiveness of BTX treatment depends on the distance between the injection site and the location of the neuromuscular junctions [5, 6], which tend to cluster in a relatively narrow band termed the innervation zone (IZ) [7]. Therefore, it is of clinical importance to determine the IZ location, as this may help to optimize BTX dosage.

Motor unit action potentials (MUAPs) propagate in opposite directions from the neuromuscular junctions toward the muscle tendons. Surface electromyography (EMG) signals recorded by linear arrays or a matrix of electrodes have been widely used to identify the IZ location in many lower and upper limb muscles [7–10]. When surface EMG signals are recorded in single differential mode during voluntary contractions (VC), the IZ can be detected based either on a reversal in signal phase between two adjacent channels along the muscle fiber, or on the minimum amplitude in a single channel.

Usually, the IZ is determined by recording submaximal to maximal surface EMG during VC. However, this approach is not possible in patients who are unable to exert the necessary force because of paralysis or poor motor control. An alternative method for IZ location is to generate forces and associated EMG (i.e., M-wave) evoked by electrical stimulation (ES) [11].

One study used both VC and ES in the tibialis anterior of adults and found no significant differences in the IZ location between the two methods [11]. However, the surface EMG signals were detected using a single-column linear array, and thus could not detect the overall IZ distribution. Hence, it is uncertain whether VC and ES methods would also have detected the same IZ location in the medial and lateral portions of the muscle. Others have reported some variations in the IZ distribution in the medial-lateral and proximal-distal directions of the biceps brachii (BB) based on VC [12, 13], but some conclude that IZ location is not altered across columns [10]. No one has addressed this issue comprehensively with ES. In the previous study of the tibialis anterior [11], IZ locations were obtained during low VC and ES force contractions only, equal to about 10% of the maximum voluntary contraction (MVC) force. Others have shown that the IZ location is dependent on the force level of the VC used to detect it [14, 15], but whether this is the case with different intensities of ES is unknown. It is possible that different groups of motor units (motor axons) with different EMG properties may be activated at low versus high stimulus intensities, with corresponding differences in the IZ location.

In this study, two matrices of electrodes were used to provide bi-dimensional spatial distribution of BB activity [16, 17]. Surface EMG signals were collected under different VC levels and ES current intensities. The purpose of the study was to assess: 1) whether the BB IZ location shifts significantly with increases in ES current intensity; and 2) whether there are significant differences in IZ location between the VC and ES methods.

## Methods

### Participants

Eight healthy men between 20 and 33 years of age (mean ± SD, 28.9 ± 4.8 years) volunteered to participate in the study. Their mean height and weight were 171.5 ± 7.5 cm and 65 ± 12 kg, respectively. None had a history of injuries to the upper limb, nor any neurological or cardiovascular complications. They were informed of the possible risks and discomfort of the experiments, and signed an informed consent approved by the local ethics committee (ethical approval number: GWIRC-AF/SC-07/2016.20).

### Instrumentation

**Fig. 1:**
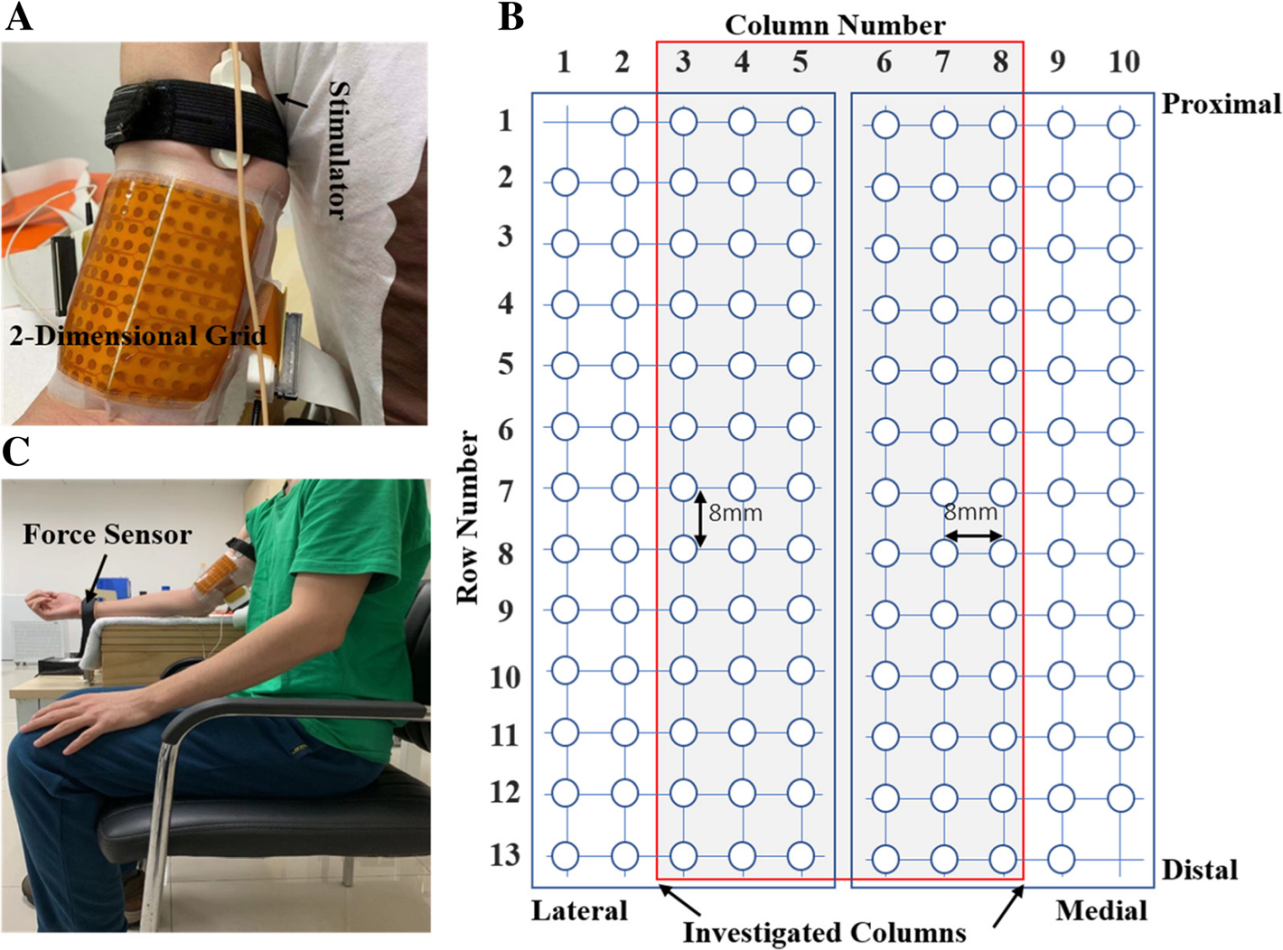
**a** High-density electrode matrix consisting of a grid with 5 columns (that are positioned parallel to the muscle fiber direction) and 13 rows. **b** Schematic representation of the two adhesive 2D matrices. **c** Illustration of the experimental setup

Two electrode matrices were used to record VC and ES surface EMG (ELSCH064NM2, Bioelettronica, Torino, Italy, Fig. 1a-b). Each electrode matrix consists of 64 electrodes with an 8 mm inter electrode distance (IED) arranged in a grid of 5 columns by 13 rows (one column consisted of 12 electrodes and the other four of 13 electrodes). A piece of double adhesive foam (1 mm thick), that contained cavities for electrode paste, was placed between the skin and matrix. Each of the cavities was filled with conductive gel to ensure proper electrode-skin contact. The VC and ES surface EMG were recorded by a signal amplifier in monopolar acquisition mode (EMG-USB2, sampling frequency of 2048 Hz, 12-bit A/D converter, Bioelettronica, Torino, Italy). The surface EMG was amplified 1000x and 100x during the VC and ES protocols, respectively.

For ES responses, a constant-current stimulator (DS7A, Digitimer, Herthfordshire, UK) and bar electrode (3 cm inter-electrode spacing) were used to evoke BB M-waves. The force during elbow flexion was sensed by a load cell (CZL-3 T, Leitai, Bengbu, China) attached to a table (Fig. 1c), and recorded by the EMG-USB2 device.

### Procedure

Each subject sat in a chair with their back fully against the backrest, with the hip, knee, and ankles joints flexed about 90°. The dominant arm (right side in all cases) was positioned in a custom-made force measuring device (Fig. 1c). The elbow angle was set at 120° degrees (180° = full extension) and the forearm was supinated. The load cell and wrist were tightly connected with an inelastic strap to measure the vertical force at the wrist resulting from contraction of the elbow flexor (BB) muscles. The height of the chair and the arm support could be adjusted for each subject individually.

The skin of each subject was first shaved and cleaned with alcohol to reduce the skin-electrode impedance. The BB was detected based on palpation during a mild contraction. The two electrode matrices were joined together, and placed so the columns were parallel to the muscle fibers, with Matrix 1 over the lateral head and Matrix 2 over the medial head of the BB (Fig. 1b). The matrices were positioned below the stimulating bar electrode, meaning that surface EMG was recorded from the distal three-quarters of the BB. The matrices were firmly fixed with elastic bandages wrapped around the upper arm. A ground electrode was placed at the elbow.

#### ES protocol

The stimulation bar electrode was positioned vertically over the musculocutaneous nerve at the most proximal region of the BB. Single pulses (1 ms duration) were applied every 5 s at progressively greater current intensity (5-mA increments) until the M-wave peak-to-peak amplitude did not increase despite further increments in current [18]. The current intensity that evoked the maximum M-wave (M_max_) was then recorded as the maximum current intensity (I_max_). Single pulses (0.2 Hz, 1 ms duration) at each of five different current intensities were then applied. The targeted current intensities were 20, 40 60, and 80% and 100% I_max_ respectively.

#### MVC and submaximal force protocol

Each subject performed two to three MVCs and the largest of the trials was adopted as the MVC value. Subjects were strongly encouraged to give their best effort and were asked to maintain a stable shoulder, elbow, and wrist position. Each subject then completed a series of 5-s submaximal contractions at 20, 40, 60, and 80% MVC respectively. For each target, subjects performed the task 2 times with a 1–2-min rest between each to avoid muscle fatigue.

Force, as well as one selected M-wave channel from the EMG-USB2 system, were recorded to a second data collection system (1401 Plus, Cambridge Electronic Design, UK) and data collection software (Spike 2, Cambridge Electronic Design), and displayed on a second monitor in front of the subject. This was necessary for more immediate on-line feedback of target forces and M-waves compared to the processing speed of the EMG-USB2 system.

### Signal processing

In some subjects, the surface EMG of the most two lateral and most two medial side columns were of low quality. The reasons might be that the electrodes of these columns were further way from the muscle compared to the more centrally located electrodes and/or poor electrode-skin contact. Therefore, these 4 columns were eliminated, leaving 6 columns for data analysis (Fig. 1b).

The monopolar signals of each column were post-processed to single differential signals and then plotted (R2017a, The MathWorks Inc., MA, USA). As reported in previous studies [7, 14], the IZ was located either as the channel with the smallest signal amplitude or between the two adjacent channels whose signals were of opposite polarity. As the IED used in this study was 8 mm, the spatial resolution for IZ determination was 4 mm.

### Statistical analysis

The distribution of data was tested using the Kolmogorov-Smirnov normality test. For both VC and ES conditions. A one-way repeated measure ANOVA was performed to determine the effect of VC level (%MVC) or ES intensities (%I_max_) on the IZ position, which was represented as the averaged row number (resolution 0.5) over all the columns. When a significant overall effect was confirmed, the Bonferroni-corrected Post-hoc test for multiple comparisons was done. The IZ row number obtained through VC and ES were compared using a Paired t-test. A *p*-value less than 0.05 was considered to be statistically significant. The analyses were performed using the SPSS software (SPSS, Chicago, IL).

## Results

**Fig. 2:**
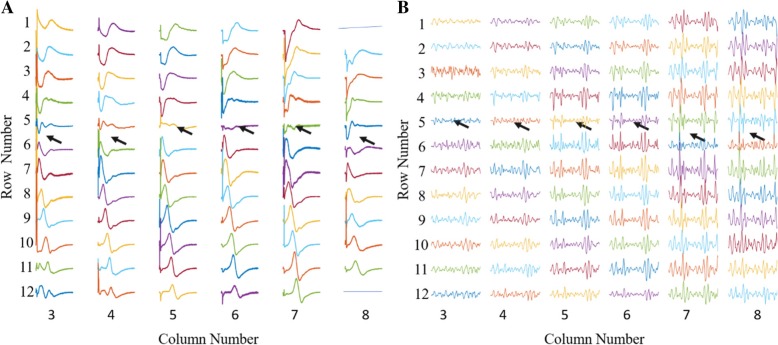
Examples of IZ locations of the BB muscle from one subject (no. 8), based on ES evoked maximal M-waves (**a**) and a VC at 60% MVC (**b**). Column 3 is the most lateral column and Row 1 is the most proximal row

**Fig. 3:**
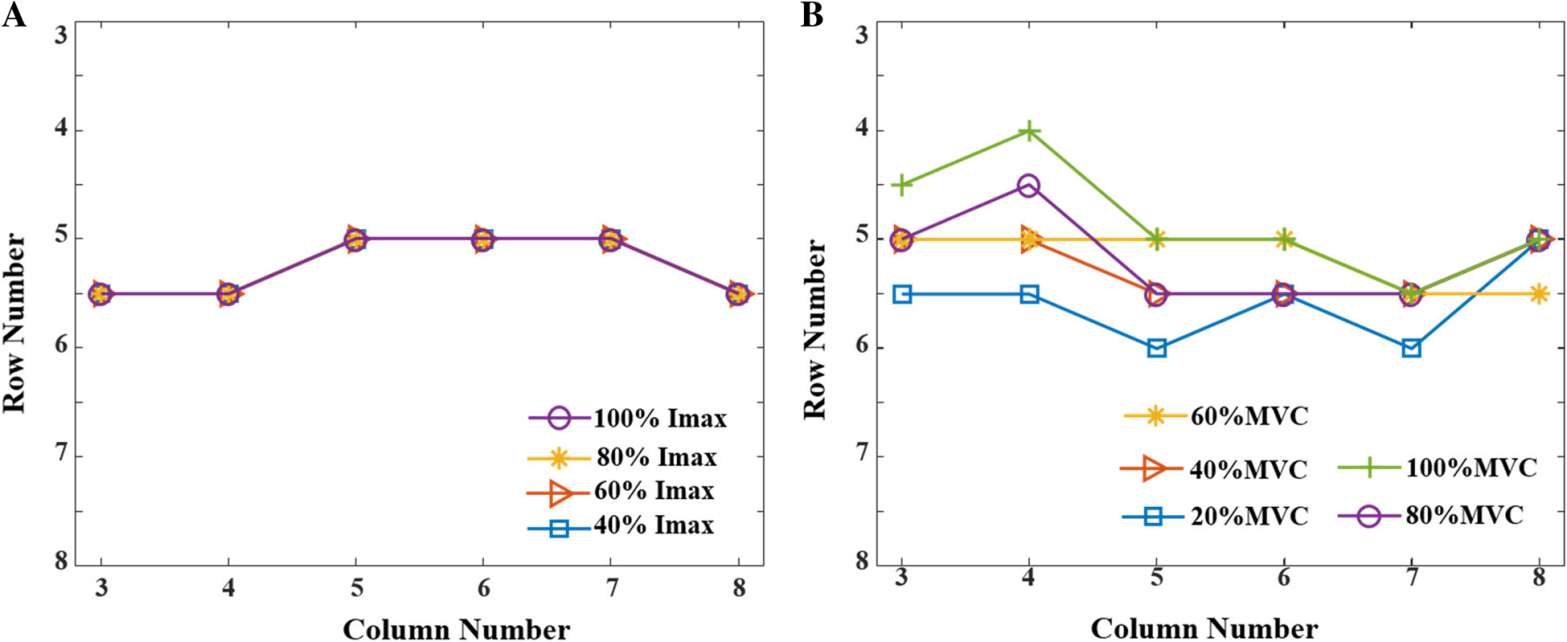
IZ location (row number) of one representative subject (no. 8) in individual columns with increasing ES (**a**) or VC level (**b**)

The IZ locations obtained through all VC and ES intensities showed normal distributions (*p* > 0.05). Typical surface EMG recordings of maximal M-waves and VC at 60% MVC are shown for one subject (no. 8) in Fig. 2a-b, respectively. In these trials, the estimated IZs (arrows) were at row 5 or between rows 5 and 6 (i.e. row 5.5) for the six columns. The IZs in each column at the different ES and VC intensities of subject 8 are also shown (Fig. 3a-b). In this subject, the IZ for all ES and VC intensities ranged from row 4 to row 6. The ES IZ within a particular column was unaffected by the stimulus intensity, but there were small IZ differences (4 mm) between columns (Fig. 3a). The VC IZ shifted proximally (up to 12 mm) with increasing MVC percentage. There were only slight IZ differences between columns (up to 8 mm or 1 row, Fig. 3b). Given this, the IZ of the muscle was represented as the averaged row number over all the columns.

### ES-derived IZ

**Fig. 4:**
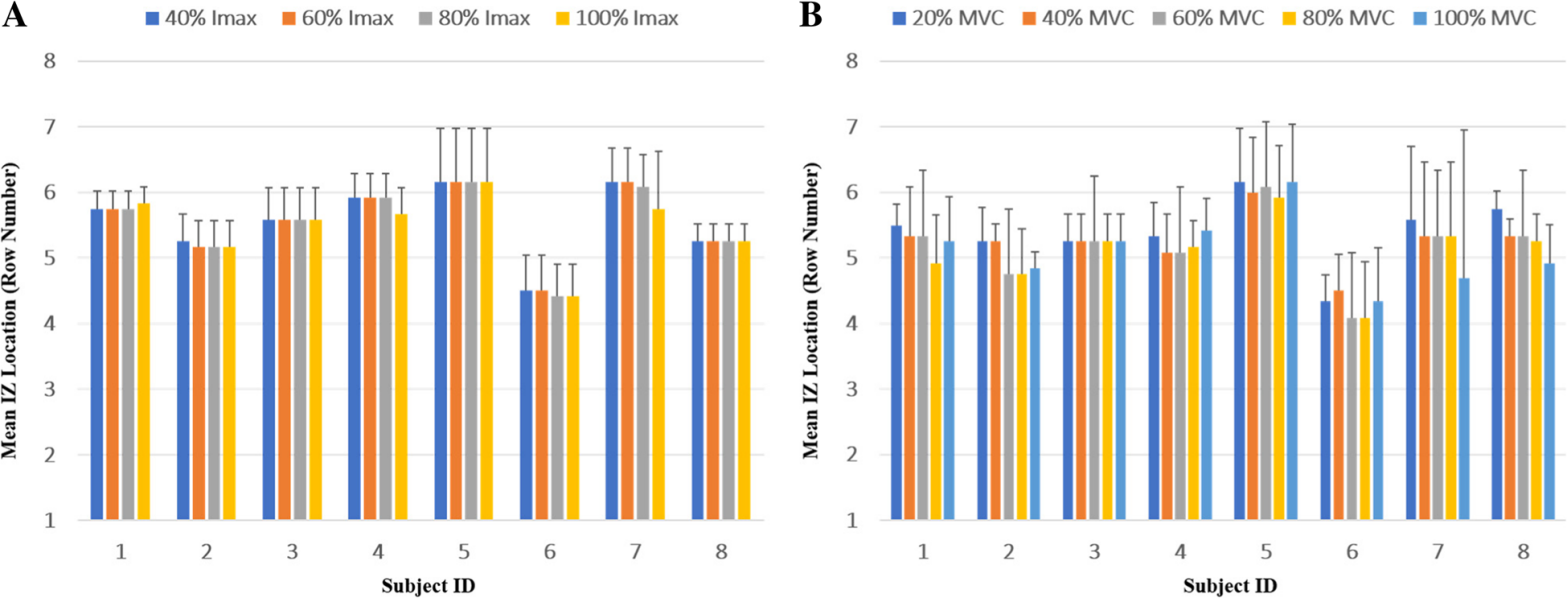
Mean (SD) IZ location (row number) of all six columns at different ES intensities (**a**) and VC levels (**b**) for each of the 8 subjects

**Fig. 5:**
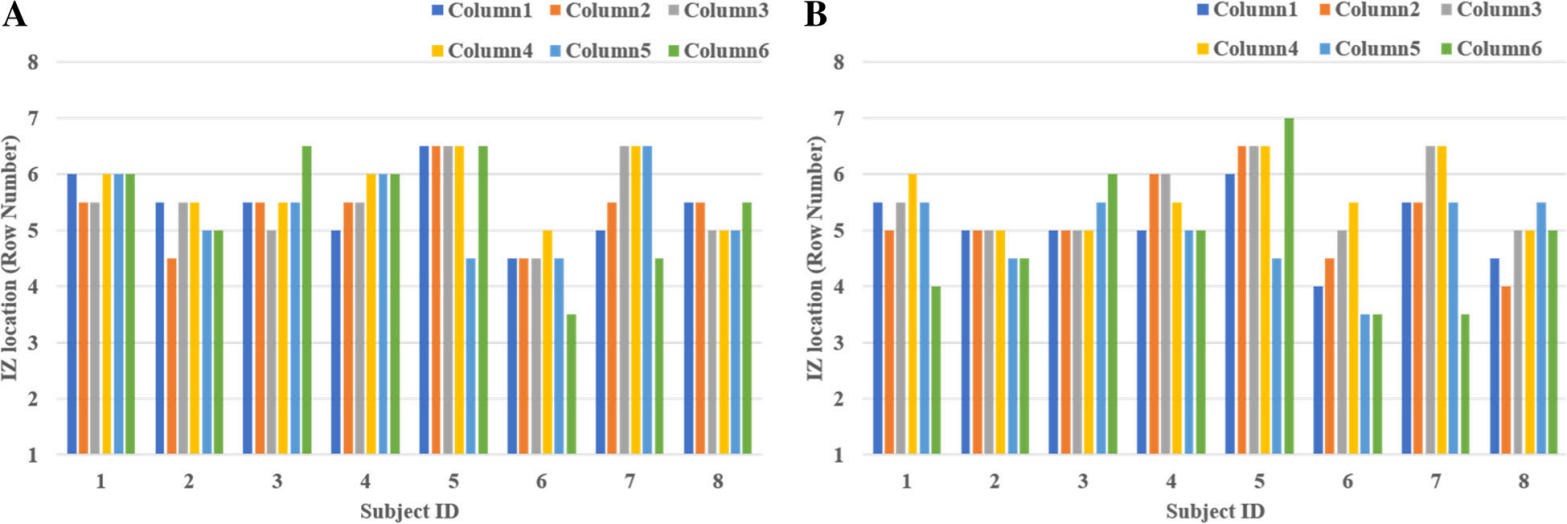
The IZ for each column at 100% I_max_ (**a**) and 100% MVC (**b**) for each subject

Some subjects had no observable M-wave at 20% I_max_. Therefore, only M-wave data from 40, 60, 80 and 100% I_max_ are reported. There were small differences in the IZ at different stimulus intensities within a subject, but the differences were not consistent across subjects. Individual IZ at the different stimulus intensities, each averaged across the 6 columns, are shown in Fig. 4a. Overall, with the medium effect size, the IZ was found to be unrelated to stimulus intensity (*p* = 0.672, partial eta square = 0.07, Table 1). The IZs for each column at 100% I_max_ are shown for each subject in Fig. 5a. There were small differences in the IZ between the different columns within a subject, but the differences were not consistent across all 8 subjects.

**Table 1:** ANOVA on IZ position with VC level (20, 40, 60, 80 and 100% MVC) or ES current intensities (40, 60, 80 and 100% Imax) as fixed factors

Method	Factors	F	p	Effect Size (partial Eta square)
*ES*	% I_max_	0.522	0.672	0.07
*VC*	% MVC	3.934	0.012	0.36

### VC-derived IZ

Figure 4b shows individual IZ at the different VC levels (20, 40, 60, 80 and 100% MVC), each averaged across the 6 columns. In most instances, the IZ shifted proximally with increasing VC level. Overall, the IZ was significantly affected by the VC level (*p* = 0.012, partial eta square = 0.36, Table 1). Post-hoc analysis indicated that the IZ was more proximal during the 60 and 80% MVC compared to the 20% MVC. The IZ was also more proximal during the 100% MVC compared to the 20% MVC, but the difference was not statistically significant. There were also differences in VC IZ between different columns within a subject, but the differences were not consistently observed across all subjects. The IZs for each column at 100% MVC are displayed for each subject in Fig. 5b.

### VC vs. ES IZ location

**Fig. 6:**
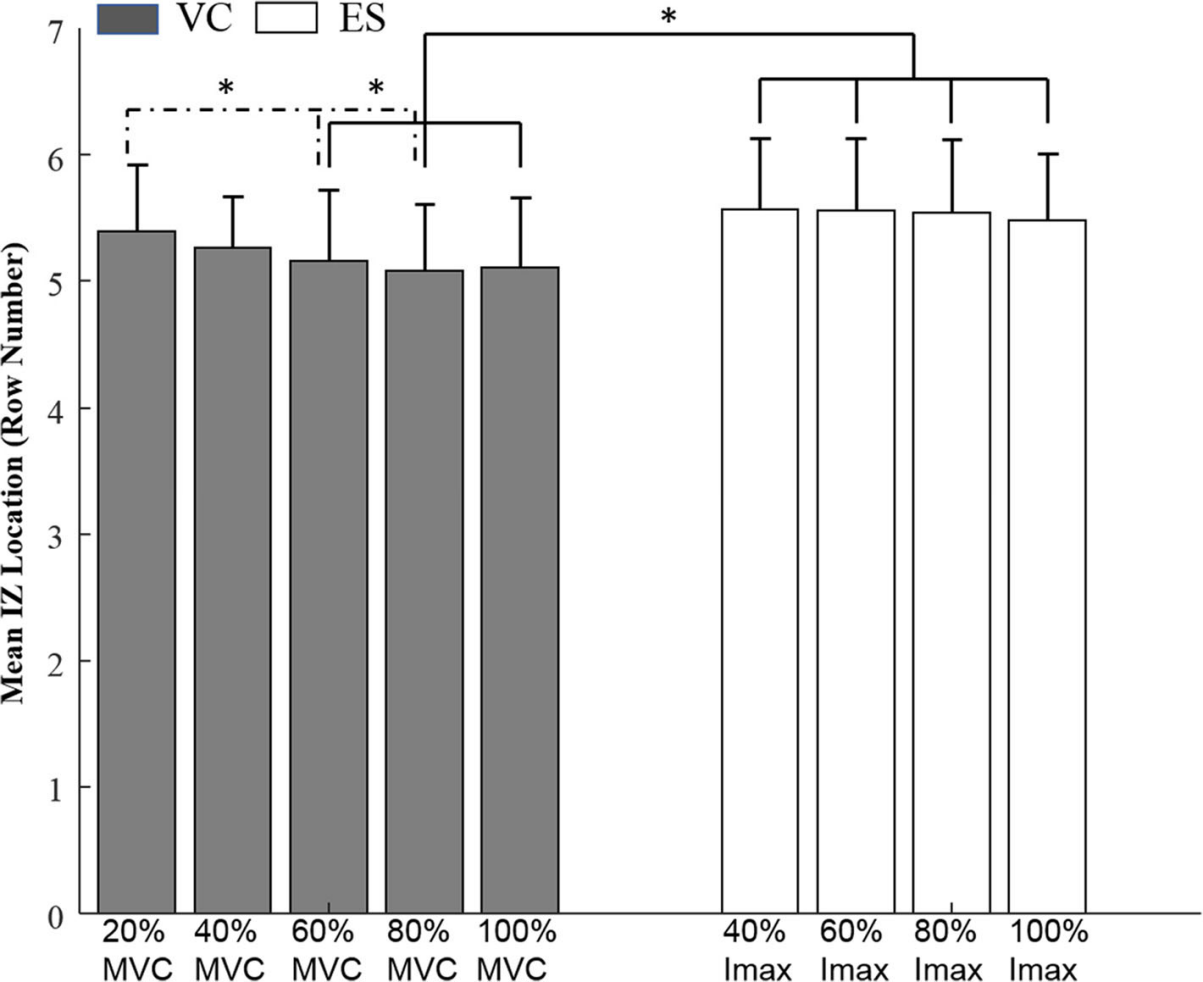
Group mean (SD) IZ of all six columns at different VC levels (grey) and different ES intensities (white). * Significant difference between ES and VC derived IZ as well as between different VC intensities (*P* < 0.05)

The mean (*N* = 8) IZs of all columns during VC and ES at the different VC levels and ES intensities are shown in Fig. 6. The IZ during the 20% MVC was similar to the ES IZ (*p* > 0.05, Table 2, paired t-test). However, the IZs for the higher VC levels were located more proximally compared to ES IZs, and the differences were significant for the three highest VC levels (60, 80, and 100% MVC).

**Table 2:** Paired t- test *p*-values and Cohen’s d values for comparison of VC and ES IZ row number at different contraction levels and stimulus intensities: *p*-value (Cohen’s d value)

Contraction Levels	40% Imax	60% Imax	80% Imax	100% Imax
20% MVC	0.351 (0.35)	0.267(0.43)	0.270(0.42)	0.270(0.42)
40% MVC	0.067(0.76)	0.060(0.79)	0.065(0.81)	0.056(0.81)
60% MVC	**0.012**(1.18)	**0.007**(1.32)	**0.006**(1.37)	**0.007**(1.37)
80% MVC	**0.007**(1.32)	**0.005**(1.45)	**0.004**(1.48)	**0.004**(1.48)
100% MVC	**0.019**(1.08)	**0.019**(1.09)	**0.015**(1.13)	**0.015**(1.13)

Entries in boldface indicate a statistically significant difference (*p* < 0.05)

## Discussion

The aim of the present study was to estimate and compare BB IZ distributions under different ES current intensities and VC levels with two electrode matrices. The BB was chosen as it plays an important role in upper limb function. It is often affected by spasticity in patients with neurological disorders, and thus is a useful model for assessing efficacy of various treatments [19].

### Detection of the IZ during ES

In the ES protocol, we examined whether the detected IZ differed between low and high stimulus intensities and between columns (see section B below). We found the IZ on average was unrelated to stimulus intensity (Fig. 4a and Table 1).

There are a number of possible reasons why the IZ was unrelated to stimulus intensity. During ES, the M-wave is largely completed before the muscle shortens. Hence, any differences in muscle shortening due to differences in stimulus intensity likely had little impact on the recorded IZ. Previously, the IZ was found to shift proximally with increasing VC levels [13, 19], and this was confirmed in the present study. This shift during VC likely reflects muscle shortening relative to the recording electrodes.

Another explanation for the lack of stimulus intensity on the IZ may relate to the recruitment order and location of different sized motor axons (motor units). During electrical nerve stimulation, progressive increases in stimulus intensity activate axons according to their size, with larger axons activated before smaller axons [20, 21], although some also found that smaller axons were activated first, similar to voluntary contractions [22–24]. In addition, for both the vastus lateralis and biceps brachii, it has been found that smaller motor units tend to be located deeper in the muscle, whereas larger motor units tend to be located more superficially [25, 26]. The IZ detected by high-density surface electrodes reflects the activity of more superficial muscle fibers [27]. With increasing ES intensity, the added recruitment of the smaller deeper MUs may have relatively little effect on the IZ location. In contrast to orderly recruitment according to axon size, some have argued that electrical nerve stimulation in-vivo activates axons randomly [28–30]. With random recruitment, the location of motor units is secondary and there may be a consistent proportional contribution of small and large motor units to the IZ, regardless of ES intensity. Our findings suggest that the IZ is independent of stimulus intensity, possibly because IZ detection was not confounded by muscle shortening and recruitment order of different sized motor units.

### IZ distribution across different columns and subjects

Both the results of the ES and VC protocols indicated that the IZ on average was unrelated to column location. This is consistent with the previous study, which found that IZ location did not change across columns [10]. However, there were some between-column differences in the IZ within individual subjects. For example, in subject 5, the IZ of column 7 is more proximal than the other columns during both VC and ES (Fig. 5). In contrast, in subject 3, the IZ of column 8 is more distal than the other columns. These individual column-related differences in IZ may reflect normal biological variability of IZ location within the medial and lateral head of the BB. Other studies also found the IZ could differ between subjects. For example, Saitou et al. [7] demonstrated that the distribution of motor unit IZs varied substantially between subjects in both upper and lower limb muscles under VC. Another study also observed that the location of the main IZ was highly variable along the BB muscle belly between subjects [14]. Botter et al. [31] investigated the uniformity of IZ location for lower limb muscles in healthy subjects using ES and showed IZ inter-individual differences.

To conclude, we have shown that a two-dimensional electrode matrix can characterize spatial differences of IZ in the medial–lateral direction of the muscle [12], unlike a single electrode array. The demonstrated inter-subject variability of the IZ is of great importance for individual determination of IZ locations prior to clinical treatments such as BTX injection.

### Concordance of IZ location between VC and ES

The average IZ during the 20 and 40% MVC was similar to the IZ derived by ES (Table 2). This is consistent with a previous study that found a similar IZ during a 10% MVC and low intensity ES of the tibialis anterior [10]. However, we found that the IZ tended to be more proximal during strong VC (60–100% MVC, Table 2) compared to ES (Figs. 4b and 6).

One explanation for the more proximal IZ location during strong VC is likely muscle shortening as mentioned above, and discussed by others [13]. During voluntary contractions, the IZ is observed while the muscle is contracted. On the contrary, during a single pulse stimulation, the IZ is observed before the muscle begins to twitch. Therefore, a more distal IZ should be expected in electrically elicited contractions. Another possible explanation for the more proximal IZ during VC compared to ES may relate to the opposite order of motor unit recruitment under the two types of contractions [24]. Hence, relatively larger superficially located motor units would be activated as VC force increased compared to ES.

### Clinical relevance

ES has advantages for estimating IZ, particularly in light of our finding that it is independent of stimulus intensity. Detecting the IZ by sub-maximal ES may be preferred for certain situations including patients with paralysis and for BTX injection. ES also revealed subtle medial-lateral differences in IZ between subjects that can be located with a matrix. This may be of practical value for optimal BTX dosage, at least for superficial fibers.

### Limitations

There are a few limitations in the present study that should be considered for further work in this area. Firstly, the IED of the two matrices used in this study was 8 mm. This distance corresponds to a spatial resolution for IZ detection of 4 mm, relatively large compared to others [11, 15]. Hence, our method may not detect potentially smaller (i.e., 2–3 mm) medial-lateral or intensity-related differences in IZ. Automatic detection algorithms and signal processing [32] could be used to locate the IZ and might improve the resolution. Secondly, the number of subjects studied was only 8, which is relatively small compared with previous studies. The partial Eta square showed that for ES, the effect size is only medium. More subjects will be helpful to precisely investigate the effect of different ES current levels on the IZ location. Thirdly, each subject was studied only once in this study. Further tests are necessary to determine repeatability of the recordings. In addition, we only investigated IZ location in healthy subjects, and it would be interesting to investigate how the findings may differ in patients with neurological disorders.

## Conclusions

We investigated two methods (VC and ES) for detecting the BB IZ in healthy subjects. The results demonstrated that: 1) there might be no effect of ES intensity on IZ location; 2) there were some spatial differences in IZ within individual subjects; 3) IZ locations were similar between ES and low-level VC. Detection of the IZ using ES and electrode arrays is non-invasive, and convenient. Complementary studies are needed to assess the IZ in patient populations. Testing the two methods of IZ detection on patients with spasticity may be useful to optimize BTX dosage.

## Data Availability

The authors confirm that all data underlying the findings are fully available without restriction. Data are available from the Guangdong Work Injury Rehabilitation Center Institutional Data Access/Ethics Committee for researchers who meet the criteria for access to human subject data.
